# Arctiin Antagonizes Triptolide-Induced Hepatotoxicity via Activation of Nrf2 Pathway

**DOI:** 10.1155/2020/2508952

**Published:** 2020-10-16

**Authors:** Yuyan Zhou, Li Xia, Weiqiang Yao, Jun Han, Guodong Wang

**Affiliations:** ^1^School of Pharmacy, Drug Research & Development Center, Wannan Medical College, Wuhu, Anhui 241002, China; ^2^Anhui Provincial Engineering Research Center for Polysaccharide Drugs, Wuhu, Anhui 241002, China; ^3^Anhui Province Key Laboratory of Active Biological Macro-molecules, Wuhu 241002, China

## Abstract

Triptolide (TP) is the most effective ingredient found in the traditional Chinese herbal *Tripterygium wilfordii* Hook F, and it is widely used in therapies of autoimmune and inflammatory disorders. However, the hepatotoxicity induced by TP has restricted its use in clinical trials. Arctiin is known as a protective agent against oxidative stress, and it exerts liver-protecting effect. This study was aimed at investigating the protective role of arctiin against TP-induced hepatotoxicity using *in vitro* and *in vivo* models. The results indicated that TP not only obviously induced liver injury in mice but also significantly inhibited the growth of HepG2 cells and increased the level of intracellular reactive oxygen. Furthermore, TP obviously decreased the expressions of proteins of Nrf2 pathway including HO-1, NQO1, and Nrf2 associated with oxidative stress pathway. However, the above experimental indexes were reversed by the treatment of arctiin. Our results suggested that arctiin could alleviate TP-induced hepatotoxicity, and the molecular mechanism is likely related to its capacity against oxidative stress.

## 1. Introduction

Triptolide (TP) is a diterpene triepoxide isolated from *Tripterygium wilfordii* Hook F, and it has been reported to have diverse pharmacological effects such as immune modulation, antiproliferative, proapoptotic, anti-inflammatory, immunosuppression, and tumor inhibition, and it has good efficacy for treating asthma, shin disease, and rheumatoid arthritis [1–3]. However, the safety window of TP is very narrow, and the effective and toxic doses are very close. The accumulated use of TP can result in severe toxicity and side effects on multiple organs especially the liver, kidney, and body system such as the bone marrow and immune and reproductive system. This has limited the clinical use of TP [4]. The mechanism of TP-induced liver damage has been largely linked to oxidative stress via the instigation of over generation of reactive oxygen species (ROS), anionic peroxides, liver apoptosis, autophagy, depletion, and inhibition of antioxidant enzymes activities [5–7]. The high levels of ROS and reactive nitrogen species (RNS) inhibit ATPase, sodium, and calcium pump activity of cell membrane leading to decrease in the mitochondrial membrane potentials (MMP) and the release of apoptosis factor caspase-3 to damage liver cells [8–13].

*Arctium lappa* L is a traditional Chinese medicinal perennial plant that has been in use for hundreds of years as a nutritive vegetable and edible traditional medicine [14]. It is used traditionally as a diuretic, carminative, anti-infection, anti-inflammatory, and as anti-TB [15]. Several chemical entities have been isolated from the plant including arctigenin and arctiin [16]. Arctiin is a lignan glycoside, found as one of the major bioactive constituent isolated from the seeds, leaves, and roots of *A. Lappa* L. [17]. Several studies have established the efficacy of arctiin as an anti-tumor, anticancer, immunoregulator, anti-inflammatory, antidiabetic, neuroprotection, and hepatoprotective agent [18–20]. Previous studies have proven the ability of arctiin to alleviate the accumulation of calcium and decline of MMP induced by elevated levels of ROS in liver cells as well as decrease the ratio of liver apoptosis resulting from oxidative stress pathway [21]. In addition, our preliminary investigation also indicated that arctiin showed potent antioxidant capacity in models of oxidative liver damage.

Recently, Keap1-Nrf2/ARE signaling pathway is considered to be the most important endogenous antioxidant stress signaling pathway, and it plays a crucial role in the body's defense against various oxidative stress-induced injuries [22, 23]. Nrf2, a cap-n-collar (CNC) regulatory protein, plays a significant role in this pathway, and it exerts wide power in various organs. Under normal physiological conditions, Nrf2 combined with its inhibitor Keap1 with nonactive state exists in the cytoplasm, and it is rapidly degraded by ubiquitin proteasome system to maintain low transcriptional activity in physiological state. Elevated levels of ROS and the other reactive nucleophile can cause the uncoupling of Nrf2 and Keap1 to trigger the activity of Nrf2. After it is transported into the nucleus, Nrf2 binds to Maf protein to form a heterogeneous dimer and then ARE protein. The trimer activates target genes expressions to regulate the activities of phase II metabolic enzymes and antioxidant enzymes, thus exerting great influence on resistance to oxidative damage. Keap1 Nrf2/ARE signaling pathway regulates more than 200 encoded endogenous genes, including antioxidation protein genes, detoxification enzymes genes, and anti-inflammatory protein genes which plays vital roles in enhancing capacities of organs against oxidative stress, inflammation, tumor, and apoptosis [24, 25]. Due to the antioxidant effect displayed by arctiin, we envisaged that it can attenuate TP-induced liver toxicity, and it may possibly exert its protective role through the Nrf2 pathway. Thus, this study investigated the hepatoprotective effect of arctiin in TP-induced hepatotoxicity.

## 2. Materials and Methods

### 2.1. Cell Culture

Human hepatoma (HepG2) cells were purchased from Jiangsu Kaiji Bio-Technology Co. Ltd (Nanjing, China). The cells were routinely cultured in Dulbecco's Modified Eagle's Medium (Hyclone, Logan, USA) supplemented with 10% fetal bovine serum, 100 U/ml penicillin, and 100 *μ*g/ml streptomycin (Gibco Life Technologies, Grand Island, NY, USA). HepG2 cells were cultured in a 37°C humidified incubator with 5% CO_2_, digested with trypsin, and allowed to pass on to the logarithm period of cells. Cells were treated with arctiin and TP. Arctiin and TP were purchased from Yuanye biological company (Shanghai, China) and were freshly dissolved in 0.1% DMSO before use.

### 2.2. MTT Assay

HepG2 cells were seeded into 96-well plate at a density of 1 × 10^4^ cells/ml. After cell adherence, cells were grouped for experiments with TP (0-320 nM) and arctiin (0-128 *μ*M) to determine an effective dose for further experiments. After the determination of an appropriate dose, cells were further treated with TP (50 nM), arctiin (50 *μ*M), and a combination of pretreatment with arctiin (50 *μ*M) for 12 h followed by TP (50 nM) treatment in order. All cell treatments were performed for 24, 48, and 72 h. After the treatment period, 20 *μ*l of MTT (5 mg/ml) was added to each well and incubated for another 4 h. The medium was removed, and DMSO (200 *μ*l) was added to each well, and the absorbance was measured at 570 nm with a microplate reader. The growth inhibition curves were evaluated by nonlinear regression analysis with SPSS 16.0 (SPSS Inc., USA).

### 2.3. Calcein-AM/PI Staining

HepG2 cells were cultured using the methods described above and treated with PBS (control), TP (50 nM), arctiin (50 *μ*M), and a combination of pretreatment with arctiin (50 *μ*M) for 12 h followed by TP (50 nM) treatment in order. After 24 h, cells were washed with PBS twice and detached with 0.25% trypsin. Cells were collected, and 1 ml of binding PBS and 0.5 ml of AM/PI double staining fluid were added in sequence. The solution was incubated in the dark for 30 min at room temperature and observed under a laser scanning confocal microscope.

### 2.4. Hoechst 33258 Staining

After cell culture and treatment with PBS, TP (50 nM), arctiin (50 *μ*M), and a combination of pretreatment with arctiin (50 *μ*M) for 12 h followed by TP (50 nM) treatment in order. After 24 h, the cells were fixed with paraformaldehyde and further incubated with Hoechst 33258 staining fluid in the dark at room temperature. After 30 min of incubation, the observed changes of the cell nucleus were observed under a laser scanning confocal microscope.

### 2.5. Apoptosis Assay

HepG2 cells were seeded in 6-well plate at a density of 1 × 10^6^ cells per well and treated with PBS (control), TP (50 nM), arctiin (50 *μ*M), and a combination of pretreatment with arctiin (50 *μ*M) for 12 h followed by TP (50 nM) treatment in order. After 24 h, the cells were washed with PBS twice, and 0.25% trypsin was used to detach the cells. Then, cells were centrifuged, and the sediment collected was used to determine the apoptosis rate with the aid of flow cytometry (BD Biosciences, CA, USA) using Annexin V-FITC/PI double staining kit.

### 2.6. Western Blot Analysis

HepG2 cells were seeded into 6-well plates at 1.0 × 10^6^ cells per well and treated with PBS (control), TP (50 nM), arctiin (50 *μ*M), and a combination of pretreatment with arctiin (50 *μ*M) for 12 h followed by TP (50 nM) treatment in order. After cell treatment for 24 h, the cells were washed twice with PBS and lysed with RIPA lysis buffer containing protease and phosphatase inhibitor cocktail (Beyotime Biotech, Haimen, China). The protein concentrations of the lysates were determined by bicinchoninic acid (BCA) protein assay kit (Beyotime Biotech, Haimen, China). Equal amounts of proteins were separated by SDS-polyacrylamide gel electrophoresis (PAGE) and transferred to PVDF membranes. After been blocked with 5% skim milk in Tris-buffered saline containing 0.2% tween-20 for 1 h at room temperature, the membranes were incubated with primary antibodies at 4°C overnight. The following primary antibodies were used: *β*-actin (1 : 5000), Nrf2 (1 : 2000), HO-1 (1 : 2000), and NQO1 (1 : 2000). Further incubation for 1 h at room temperature with secondary antibody conjugated with horseradish peroxidase (HRP) was conducted. The membranes were visualized under ECL and analyzed using the Image J software.

### 2.7. Animals and Experiment Treatments

BALB/C mice (18-20 g, 6-8 weeks of age) were purchased from the Laboratory Animal Center of Wannan Medical College. All the mice were maintained in the pathogen-free conditions (22°C, 12 h of light, and 12 h of dark cycle) and given free access to food and water. All procedures involving laboratory animal study were in accordance with the Guide for the Care and Use of Laboratory Animals. All protocols were submitted and validated by the Ethics Committee for the Use of Laboratory Animals, Wannan Medical College. To analyze the protective effect of arctiin in TP-induced hepatotoxicity, 40 mice were randomly divided into four groups (*n* = 10/group) and designated as follows:

*Group 1*: control group orally administered with 0.9% saline solution

*Group 2*: TP group was orally administered with TP at the dose of 600 *μ*g/kg (Wang et al. 2014)

*Group 3*: arctiin was orally administered with arctiin at the dose of 500 mg/kg

*Group 4*: arctiin + TP group orally administered with TP at the dose of 600 *μ*g/kg and arctiin at the dose of 500 mg/kg

The mice were orally treated with arctiin 12 hours before the administration of TP. After 24 h, blood samples were obtained from the mice by retro-orbital plexus. The serum obtained from the blood after centrifuging was used for biochemical analysis. The mice were sacrificed, and the livers were exercised, weighed, and used for further analysis. The doses of arctiin and TP used were based on our preliminary experimental results. The body weights of mice were recorded to calculate the liver index using the following formula: liver index = liver weight/body weight.

### 2.8. Evaluation of Liver Function Enzymes

Blood samples obtained were centrifuged at 3000 rpm for 10 min at 4°C, and the serum obtained was used for estimating liver function enzymes including alanine aminotransferase (ALT) and aspartate aminotransferase (AST) using assay kits from Jiancheng Bioengineering Institute (Nanjing, China).

### 2.9. Histopathology

Liver tissues from each mouse were immersed in a formaldehyde solution composed of 10% of 37-40% formaldehyde and 90% of PBS (0.01 M, pH 7.4) for 24 h, then transferred to 70% ethanol. After fixation, the liver samples were embedded in paraffin and cut into 3 *μ*m sections, then stained with hematoxylin and eosin (H&E) for morphological evaluation.

### 2.10. Evaluation of ROS Levels in the Liver

In order to determine the Reactive Oxygen Species (ROS) levels in each group, the liver tissues were homogenized in ice cold PBS, and the homogenate was centrifuged at 3000 rpm for 10 min at 4°C. The supernatant obtained was used for measuring the ROS levels using commercially available assay kit according to the instructions of the manufacturer. In brief, the supernatants were incubated for 60 min at room temperature with 2´, 7´-dichlorofluorescein diacetate (DCHF-DA) which can be rapidly oxidized to form highly fluorescent derivative dichlorofluorescein (DCF) in the presence of ROS. The DCF fluorescence intensity was measured with a fluorescence microplate reader (excitation wavelength of 500 nm and emission wavelength of 525 nm).

### 2.11. Evaluation of Nrf2, HO-1, and NQO1 Levels in the Liver

Liver tissues of experimental animals were cut up and homogenized at 4°C after treating with the tested drugs; then, liver samples were lysed with RIPA buffer. Total proteins were obtained from lysate centrifuged at 12000 rpm for 20 min at 4°C. The protein concentrations were measured by BCA Protein Assay Kit, and equivalent amounts of protein were separated by 10% SDS-PAGE and transferred to PVDF membranes. The membranes are incubated with primary antibodies of *β*-actin, Nrf2, HO-1, and NQO1, and secondary antibodies are conjugated with horseradish peroxidase (HRP), followed by ECL detection and quantification using the Image J software.

### 2.12. Statistical Analysis

Data were expressed as mean ± SD. All statistical analysis was performed with SPSS 16.0 (SPSS Inc., USA). Differences of data were analyzed by one-way ANOVA, followed by Dunnett's post hoc test. **P** values < 0.05 were considered statistically significant.

## 3. Results

### 3.1. Effects of TP and Arctiin on HepG2 Cell Viability

**Figure 1:**
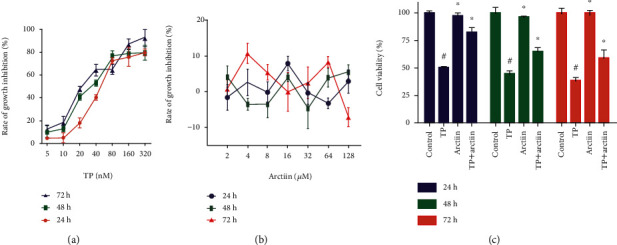
Cytotoxity of TP, arctiin, and TP + arctiin in HepG2 cells. (a) HepG2 cells were treated with triptolide (TP) at various concentrations for 24, 48, and 72 h. (b) HepG2 cells were treated with arctiin at various concentrations for 24, 48, and 72 h. (c) Cells were exposed to TP, arctiin, and TP + arctiin, and cell growth inhibition rates were measured by MTT assay. The growth inhibition rate of the cells was expressed as %. All data are represented as the mean ± SD (*n* = 6). #*P* < 0.05 compared to the control group; ^∗^*P* < 0.05 compared to the TP-treated group.

As shown in Figures 1(a)–1(c), after treatment of cells with TP, arctiin or a combination of both for 24, 48, and 72 h, the growth of HepG2 cells was obviously inhibited by TP, and the inhibition rate increased with time and concentration (Figure 1(a)), whereas this trend was not observed in arctiin-treated cells (Figure 1(b)). However, the growth inhibition rate of HepG2 cells was decreased by combination of TP and arctiin in contrast to cells treated with only TP (Figure 1(c)).

### 3.2. Effects of TP, Arctiin, or Combination on Morphological Changes of HepG2 Cells

**Figure 2:**
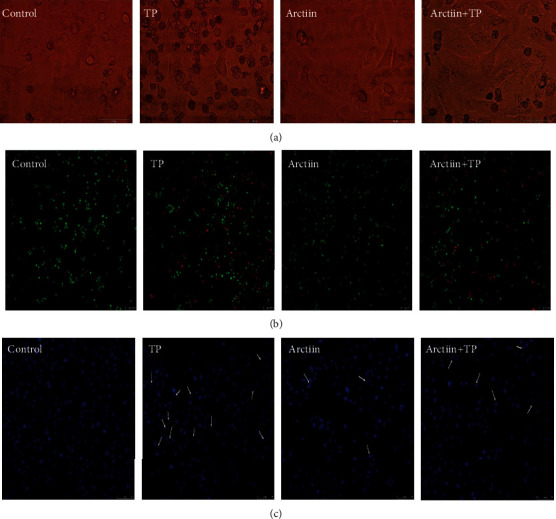
Effects of TP, arctiin, and TP + arctiin on the morphology of HepG2 cells. Treated cells were visualized using (a) light microscope and fluorescence microscope after stained with (b) Calcein-AM/PI mixed dye and (c) Hoechst 33258. White arrows indicated the condensation and fragmentation of chromatin.

As shown in Figures 2(a)–2(c), control cells were polygonal and integrally adhered to the wall, while HepG2 cells shrunk and were smaller size, and the number of adherent cells decreased significantly in the TP-treated cells when compared with the control group (PBS-treated cells). These results indicated that the TP-treated cells fully corresponded to the morphological characteristics and changes observed in cell models of apoptosis. However, arctiin reversed TP-induced apoptotic trends of HepG2 cells. In cells treated with TP and arctiin, a significant decrease in HepG2 cells apoptosis was observed as well as an increase in the number of adherent cells (Figure 2(a)).

Furthermore, we differentiated living cells, apoptotic cells, and necrotic cells under a laser confocal microscope on the basis of the results obtained from staining of Calcein-AM/PI kit; AM is the fluorescent probe of living cells while PI is indicative of dead cells. The number of red fluorescence in TP-treated cells was observed to be significantly increased when compared with the control group (PBS-treated cells), while arctiin reversed this trend (Figure 2(b)). In addition, it was observed that the nuclei of the control group had uniform size, complete morphology, and emitted even light blue fluorescence. However, the nuclei of TP-treated cells displayed some aberrant changes such as shrinkage, smaller size which resulted from chromatic agglutination, fragmentation of nucleus, and formation of apoptotic bodies. However, arctiin significantly reversed these changes (Figure 2(c)).

### 3.3. Effects of TP and Arctiin on HepG2 Cell Apoptosis

**Figure 3:**
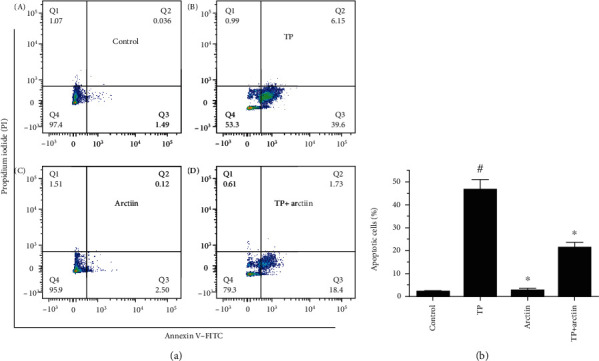
Effects of TP, arctiin, and TP + arctiin on HepG2 cell apoptosis. HepG2 cells were treated with TP, arctiin, and TP + arctiin for 24 h, and cell apoptosis was determined by flow cytometry using Annexin V-FITC/PI double staining kits. The cell populations shown in (D) represents early apoptosis, while (B) represents late apoptosis and (A) represents necrotic cells. Histograms represent apoptosis rate (%). #*P* < 0.05 compared to the control group; ^∗^*P* < 0.05 compared to the TP-treated group.

The results from the flow cytometry analysis are shown in Figure 3; the exposure of cells to TP triggered significant apoptosis in HepG2 cells (45.7% apoptotic rate) when compared to PBS-treated control cells (1.5% apoptotic rate). In contrast, the coincubation of HepG2 cells with arctiin led to an evident suppression of HepG2 cells apoptosis (20.1% apoptotic rate). These results suggested that arctiin could protect HepG2 cells from TP-induced cell apoptosis.

### 3.4. Effects of Arctiin on HO-1, NQO1, and Nrf2 when Copretreated with TP

**Figure 4:**
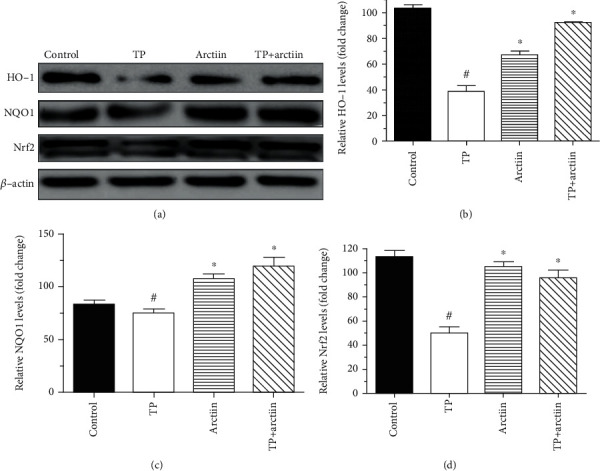
Effects of TP and arctiin on Nrf2 pathway in HepG2 cells. HepG2 cells were treated with TP, arctiin, and TP + arctiin for 24 h, and the expression levels of Nrf2, HO-1, and NQO1 protein were determined by western blot analysis. (a) Panel shows representative bands of HO-1, NQO1, and Nrf2. Histograms represent optical density values of (b) HO-1, (c) NQO1, and (d) Nrf2 normalized to the corresponding *β*-actin. Data were presented as mean ± SD (*n* = 3). #*P* < 0.05 compared with the control group; ^∗^*P* < 0.05 compared with the TP group.

As shown in Figures 4(a)–4(d), TP treatment obviously decreased the expression of HO-1, NQO1, and Nrf2, whereas, the results obtained indicated that compared with TP treated cells, the combination of TP and arctiin showed pronounced increase in the expression of HO-1, NQO1, and Nrf2.

### 3.5. Effects of TP, Arctiin, or Combination on Liver Function Enzymes and Liver Index

**Figure 5:**
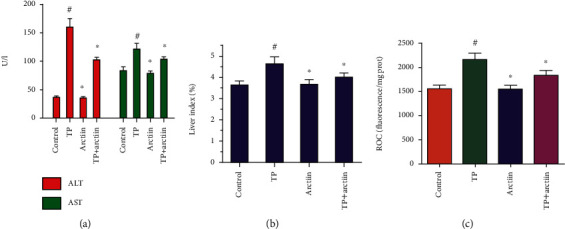
Effects of TP, arctiin, or combination on (a) AST and ALT levels, (b) liver index, and (c) ROS levels. Data were presented as means ± SD (*n* = 10). #*P* < 0.05 compared with the control group, ^∗^*P* < 0.05 compared with the TP group.

As indicated in Figure 5(a), the TP group had significantly higher levels of ALT and AST when compared to the normal control group. The group treated with arctiin had no changes in the level of ALT and AST when compared to the control group, whereas the combination of arctiin and TP significantly decreased ALT and AST levels when compared to the TP group (Figure 5(a)). Furthermore, the liver index of the TP group was markedly increased when compared to the normal control group, and the combination of arctiin and TP significantly reduced the liver index (Figure 5(b)).

### 3.6. Effects of TP, Arctiin, or Combination on ROS Levels

Compared with the control group, the fluorescence intensity indicating ROS levels in the TP group was significantly increased when compared to the control group. However, combination treatment of arctiin and TP obviously decreased the fluorescence intensity compared with the TP group (Figure 5(c)).

### 3.7. Effects of TP, Arctiin, or Combination on Morphological Changes of HE Stained

**Figure 6:**
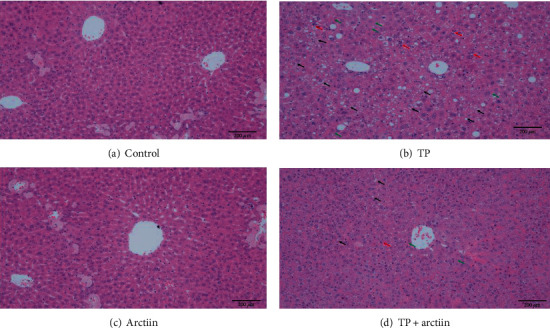
Effects of TP, arctiin, and TP + arctiin on the photomicrographs of hematoxylin and eosin-stained liver sections. Representative photomicrographs of H&E staining for liver sections from each group, respectively (bar = 200 *μ*m): (a) control group, (b) TP group, (c) arctiin group, and (d) TP+ arctiin group.

Histopathological analysis of the livers showed severe hepatocellular necrosis, extensive lipid droplets congestion, and large areas of hydropic degeneration occurred in the mice treated with triptolide alone but not in the control animals (Figures 6(a) and 6(b)). The severity of liver injury was significantly decreased in the combination of the arctiin and TP treatment group (Figure 6(d)).

### 3.8. Effects of Arctiin on HO-1, NQO1, and Nrf2 when Copretreated with TP in Mice Livers

**Figure 7:**
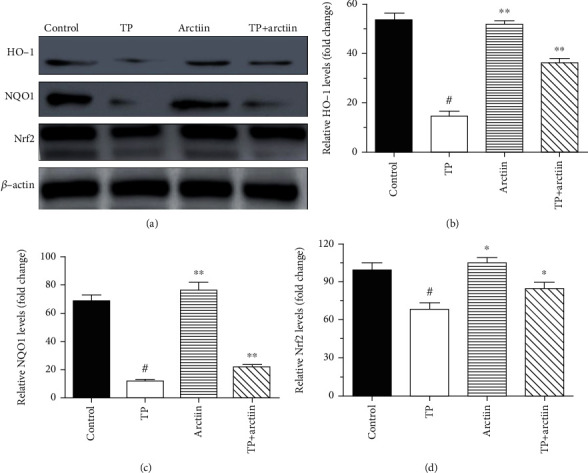
Effects of TP and arctiin on Nrf2 pathway in the livers. After the treatments (TP, arctiin, and TP + arctiin) in different groups, liver tissues of experimental animals were collected, and the expression levels of Nrf2, HO-1, and NQO1 proteins were determined by western blot analysis. (a) Panel shows representative bands of HO-1, NQO1, and Nrf2. Histograms represent optical density values of (b) HO-1, (c) NQO1, and (d) Nrf2 normalized to the corresponding *β*-actin. Data were presented as mean ± SD (*n* = 3). #*P* < 0.05 compared with the control group; ^∗^*P* < 0.05 compared with the TP group.

As presented in Figures 7(a)–7(d), TP-treated mice showed a slighter decline level of HO-1, NQO1, and Nrf2 compared with control. And these proteins were significantly upregulated with the combination of TP and arctiin.

## 4. Discussion

Although extensive researches about TP-induced hepatotoxicity have been carried out, the underlying molecular mechanisms have not yet been fully elucidated [26]. Recent researches have shown that the excessive ROS expression can evidently lead to an imbalance between oxidation and antioxidation, and it is primarily involved in the pathophysiology of TP-induced hepatotoxicity. Over generation of ROS resulted from TP stimulation can damage cellular components such as DNA, RNA, and proteins which leads to cell apoptosis. In order to enhance the efficacy and decrease its toxicity, TP is usually used in combination with other Chinese herbs or active components. As one of the main active component found in *T. wilfordii*, arctiin is commonly used in traditional Chinese medicine, and its pharmacological effect has been extensively investigated, especially its resistance to oxidative stress [27–29].

Nrf2 pathway is one of the main cellular response to toxin or chemical incited ROS and oxidative stress. It is evidently activated during cellular redox imbalance, and it protects cell damages by enhancing antioxidant capacity, metabolizing xenobiotic, and enhancing the activities of cytoprotective enzymes [30]. In addition, recent studies have indicated that Nrf2 is a key transcription factor of cell antioxidative stress system, and it plays a critical role in preventing oxidative injury. Nrf2 is involved in the transcription of downstream phase II detoxification enzymes, phase III drug transporters, and antioxidant enzymes so as to reduce cell injuries resulting from reactive oxygen [31, 32]. There are three core components in the Nrf2 pathway including Nrf2, ARE, and Keap1. In normal physiological state, Keap1, a cytoplasmic protein, combines with Nrf2 to form Keap1-Nrf2 complex which is anchored in the cytoplasmic actin of cytoskeleton, making Nrf2 too stable to enter the nucleus; meanwhile, the transcriptions mediated by Nrf2 protein are inhibited. In addition, Keap1-Nrf2 complex is a suitable substrate for U3 ubiquitin ligase promoting degradation of Nrf2 protein via ubiquitination and making it inactive. However, in conditions of excessive generation of ROS and oxidative stress, Nrf2 protein is dissociated from Keap1-Nrf2 complex and is released as a free state. The cytoplasmic free Nrf2 proteins can enter cell nucleus to initiate the Nrf2 pathway [33, 34]. During oxidative stress, oxidizing agent and electrophilic compounds bind to the cysteine residues of Keap1 to change its conformation and uncouple with Nrf2. The degradation of Nrf2 protein via U3 ubiquitin ligase is decreased, and excessive Nrf2 proteins are transposed into the nucleus, followed by recognition and combination with ARE. The results from our study indicated that the Nrf2/ARE pathway played a critical role in the protection of arctiin against TP-induced hepatotoxicity. TP inhibited the activation of Nrf2; however, our results showed that arctiin could antagonize TP-induced inhibition, thus activating further activation of the pathway to initiate antioxidant protection.

ARE is a specific DNA-promoter binding sequence located upstream of the protective genes, and it is a key link in the expression of some important antioxidases such as NQO1 and HO-1 involved in the progress of antioxidative stress and detoxification. HO-1 is a rate limiting enzyme which is responsible for catalyzing oxidative degradation of heme to produce carbon monoxide, biliverdin, and free iron which are speculated to be closely related to its antioxidative and anti-inflammatory functions [35, 36]. While NQO1 is cytosolic flavoprotein with an obligate two-electron reductase that is involved in chemo protection, detoxification reactions, and antioxidant defense via the generation of antioxidant forms of ubiquinone and vitamin E. However, overexpression and accumulation of Nrf2 in cell nucleus damage cells as well, so Nrf2 is quickly uncoupled with ARE and degraded by ubiquitination after activation [37]. Interestingly, our study indicated that arctiin activation of the Nrf2/ARE pathway evidently increased the expression of downstream genes NQO1 and HO-1, thus indicating the vital role of its antioxidant protection.

Several pharmacological properties have been attributed to arctiin including many physiological activities such as anti-inflammatory, ion, antimicrobial, antiallergic, and anticarcinogenic activities. Traditionally, arctiin is used for the treatment of liver diseases, diabetes, eczema, and skin infection [38]. Recent studies have emphasized the protective effect of arctiin in several disease models including has also been found to exert functions in protecting against endothelial cell damage, anti-Alzheimer' disease and antiaging, the antihair loss, and so on, and these bioactivities are closely related to the antioxidative stress effect of arctiin. Arctiin also protected against cardiac hypertrophy, and LPS induced acute lung injury via the inhibition of MAPKs/AKT and PI3K/AKT signaling pathways, respectively [39, 40]. Although several researches have demonstrated that arctiin could play a vital role in strong response to oxidative stress, however, there are no reported studies on the protective effect of arctiin through the study in Nrf2 pathway, an important pathway of oxidative stress, have not been reported. Our results demonstrated that arctiin could alleviate or protect against TP-induced hepatotoxicity by acting as an upstream activator to regulate transcriptional activity of Nrf2 and its downstream target (HO-1 and NQO1). The molecular mechanism may be associated with inhibiting Keap1, increasing Nrf2 translocation, and inducing the expression of target genes. In the further research, more in-depth experiments including Nrf2 nuclear translocation detection, silence of Nrf2 gene in HepG2 cells, or combined with gene knockout mouse are necessary to elucidate the mechanisms of hepatoprotective activity by arctiin.

## 5. Conclusion

In conclusion, the results from this present study suggested that arctiin exerted antioxidative effect which could alleviate TP-induced hepatotoxicity, and this antioxidant effect is mediated through the Nrf2/ARE pathway.

## Data Availability

The data used to support the findings of this study are available from the corresponding author upon request.
